# Plasma Protein Biomarker Candidates for Myelodysplastic Syndrome Subgroups

**DOI:** 10.1155/2015/209745

**Published:** 2015-09-13

**Authors:** Pavel Majek, Klara Pecankova, Jaroslav Cermak, Jan E. Dyr

**Affiliations:** ^1^Department of Biochemistry, Institute of Hematology and Blood Transfusion, U Nemocnice 1, 128 20 Prague 2, Czech Republic; ^2^Clinical Department, Institute of Hematology and Blood Transfusion, U Nemocnice 1, 128 20 Prague 2, Czech Republic

## Abstract

In recent years the plasma proteomes of several different myelodysplastic syndrome (MDS) subgroups have been investigated and compared with those of healthy donors. However, the resulting data do not facilitate a direct and statistical comparison of the changes among the different MDS subgroups that would be useful for the selection and proposal of diagnostic biomarker candidates. The aim of this work was to identify plasma protein biomarker candidates for different MDS subgroups by reanalyzing the proteomic data of four MDS subgroups: refractory cytopenia with multilineage dysplasia (RCMD), refractory anemia or refractory anemia with ringed sideroblasts (RA-RARS), refractory anemia with excess blasts subtype 1 (RAEB-1), and refractory anemia with excess blasts subtype 2 (RAEB-2). Reanalysis of a total of 47 MDS patients revealed biomarker candidates, with alpha-2-HS-glycoprotein and leucine-rich alpha-2-glycoprotein as the most promising candidates.

## 1. Introduction

Myelodysplastic syndrome (MDS) is a group of heterogeneous oncohematological bone marrow disorders characterized by peripheral blood cytopenias, ineffective hematopoiesis, bone marrow hypercellularity, and so forth [1]. MDS classification covers a range from low-risk subgroups with good patient outlook and survival, to high-risk subgroups characterized by a progression of the disease toward acute myeloid leukemia and a poor outcome [2, 3]. The molecular mechanisms that lead to the genesis of MDS and its development are not yet fully understood. Moreover, our knowledge of the changes occurring in MDS remains limited. Some findings at the DNA (chromosomal aberrations [4], up- or downregulation of genes [5], DNA methylation changes [6], single nucleotide polymorphisms [7], etc.) and RNA levels (altered expression of microRNAs in CD34+ cells [8, 9]) have been observed; however, there is a lack of detailed characterization of the changes at the protein level. Protein changes, whether in protein levels or posttranslational modifications, are expected to play a crucial role in the modern diagnostic toolkit. Considerable effort has been expended in the preparation of such tools in recent years (from the studies of plasma protein interactions with antifouling surfaces [10] to the preparation of low- or even nonfouling surfaces suitable for biochip construction [11, 12]); the topic of clinical applications in oncohematology has been reviewed by Fracchiolla et al. [13]; however, the first step has to be the identification of protein biomarker candidates. In our previous studies, we used a proteomic approach to investigate plasma proteome changes in the different MDS subgroups, covering the range from low- to high-risk subgroups: refractory cytopenia with multilineage dysplasia [14], refractory anemia with excess blasts subtype 1 [15], refractory anemia and refractory anemia with ringed sideroblasts [16], and refractory anemia with excess blasts subtype 2 [17]. Several proteins were proposed as potential biomarkers of different MDS subgroups in comparison with control groups of healthy donors. Although the control group study designs were kept similar to maintain consistency in the interpretation of the results and to facilitate comparison of the changes among the different MDS subgroups, only rough estimation may be obtained on this basis. Moreover, some criteria (statistical significance) cannot be estimated by this method at all. Therefore, the goal of this work was to reanalyze the data from our four proteomic studies of different MDS subgroups in order to evaluate the protein biomarker candidates of these different MDS subgroups.

## 2. Methods

In this work, the data from four previous proteomic studies of different MDS subgroups has been reanalyzed: refractory cytopenia with multilineage dysplasia [14], refractory anemia with excess blasts subtype 1 [15], refractory anemia and refractory anemia with ringed sideroblasts [16], and refractory anemia with excess blasts subtype 2 [17]; only patient data (no healthy control donors) were used. There were 47 myelodysplastic syndrome patients: 22 patients with refractory cytopenia with multilineage dysplasia (RCMD), 10 patients with refractory anemia or refractory anemia with ringed sideroblasts (RA-RARS), 7 patients with refractory anemia with excess blasts subtype 1 (RAEB-1), and 8 patients with refractory anemia with excess blasts subtype 2 (RAEB-2). The median of age was 57, 71.5, 68, and 63.5 years, and the patient make-up was 50%, 40%, 57%, and 38% male in RCMD, RA-RARS, RAEB-1, and RAEB-2, respectively. Patients' characteristics are summarized in Table 1. Diagnoses were established according to the WHO classification criteria [2]. All individuals tested agreed to participate in the study on the basis of an informed consent. All samples were obtained and analyzed in accordance with the Ethical Committee regulations of the Institute of Hematology and Blood Transfusion in Prague.

Scanned gel images obtained from our previous four proteomic studies were used in this study; blood collection, sample preparation, high-abundance plasma protein depletion, 2D SDS-PAGE protein separation, protein visualization, and gel digitization have been described in detail [14, 18]. Digitized gel images were processed with Progenesis SameSpots software (Nonlinear Dynamics, Newcastle upon Tyne, UK); images were divided into four groups according to MDS diagnoses, and the fold and *P* values of all spots were computed by the software using one-way ANOVA analysis. Protein identification was performed for spots (proteins within the spots) that were not submitted for protein identification in the previous studies. An HCT ultra ion-trap mass spectrometer with nanoelectrospray ionization (Bruker Daltonics, Bremen, Germany) coupled to a nanoLC system UltiMate 3000 (Dionex, Sunnydale, CA, USA) was used to perform MS analysis. Mascot (Matrix Science, London, UK) was used for database searching (Swiss-Prot). Two unique peptides (with a higher Mascot score than the minimum for identification, *P* < 0.05) were necessary to identify a protein. The procedure was described in detail previously [18]. Western blot analysis was performed as previously described in detail [19]. Briefly, 6 samples (3 males and 3 females) were used for each MDS subgroup as a pooled sample. Proteins of pooled samples were precipitated with acetone, protein pellets were diluted in a sample buffer, and SDS-PAGE was performed, followed by protein transfer to a PVDF membrane. The following primary antibodies were used: monoclonal mouse anti-leucine-rich alpha-2-glycoprotein (ab57992), 1 : 400 (Abcam, Cambridge, UK); monoclonal mouse antialbumin (A6684), 1 : 2000 (Sigma-Aldrich, Prague, Czech Republic); polyclonal rabbit anti-alpha-2-HS-glycoprotein (ab112528), 1 : 1000 (Abcam); and polyclonal mouse antiapolipoprotein A-I (H00000335-B01P), 1 : 1000 (Abnova, Taipei, Taiwan). The following secondary antibodies were utilized: rabbit anti-mouse IgG antibody conjugated with peroxidase (A9044), 1 : 80000 (Sigma-Aldrich) and goat anti-rabbit IgG antibody conjugated with peroxidase (A0545), 1 : 80000 (Sigma-Aldrich). Protein bands were visualized using a 1-Step Ultra TMB-Blotting Solution (Thermo Scientific, Waltham, MA, USA).

## 3. Results and Discussion

**Figure 1:**
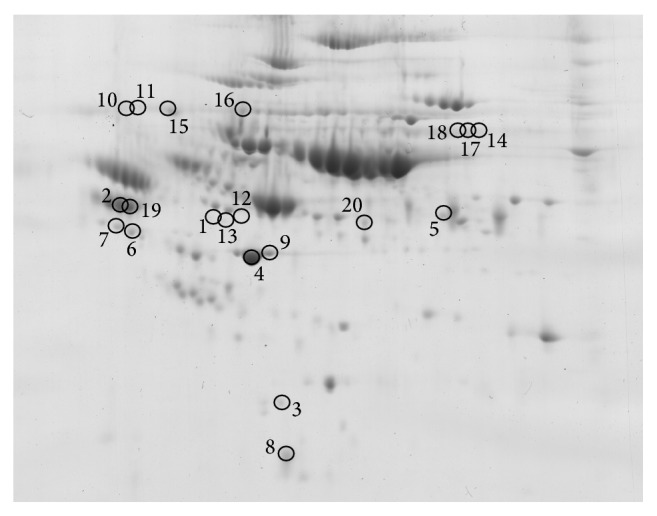
Positions of the spots. Positions of the spots with identified proteins were displayed on an illustrative 2D gel of a patient sample. For better clarity the gel image is shown as highlighted by brightness and contrast image adjustment.

The aim of this work was to evaluate plasma protein biomarker candidates of myelodysplastic syndrome subgroups by reanalyzing previously published proteomic data to allow direct and statistical comparisons. In order to select the most promising protein candidates, a two-step selection process was applied. In the first step, all four MDS subgroups were compared together and spots that were found to significantly differ (ANOVA *P* < 0.05) among the groups were selected; 42 different spots were found. In the second step, all the groups were compared mutually (each to each other), and the only spots selected in the first step were considered. In order to maintain the same level of significance, a Bonferroni correction was applied in the second step selection [20]. Therefore, the *P* value threshold for the second step comparison was lowered to *P* < 0.00833. As an additional criterion, only spots with at least a 50% change of their normalized spot volumes were accepted. There were then 23 different spots found which satisfied these criteria; proteins in 20 spots (Figure 1) were identified by mass spectrometry. The numbers of spots that were found to differ between the compared pairs of MDS subgroups are summarized in Table 2. A brief characterization of these spots (*P* value and the relative change between the groups) with regard to the compared pairs of MDS subgroups is shown in Table 3. Identification of the proteins, together with the number of unique identified peptides, accession numbers, and protein sequence coverage, is summarized in Table 4. From the results in Table 2 there is no direct correlation between the number of differences and the severity of the subgroups. Although the patient cohort is relatively small, the results support the notion proposed in our previous studies that “a degree of change” is the principal factor affecting proteome alterations observed for different MDS subgroups [16, 17]. Therefore, when protein posttranslational modifications are taken into consideration, it is not surprising that several proteins found to differ in this work were also identified to differentiate between MDS subgroups and the healthy control groups: alpha-2-HS-glycoprotein, leucine-rich alpha-2-glycoprotein, retinol-binding protein 4, hemopexin, apolipoprotein A-I, and so forth. These observations suggest that it is unlikely to find a single protein as a diagnostic MDS biomarker when only considering its plasma level change. However, finding a single protein biomarker with respect to its plasma level change can be possible for potential prognostic MDS biomarkers, as previously indicated for alpha-2-HS-glycoprotein [16, 17]. This protein seemed to decrease its plasma level relative to the severity of the MDS subgroups studied; and moreover, it was also shown that its plasma level decrease reflected the degree of malignancy found in other different tumor types [21].

**Figure 2:**
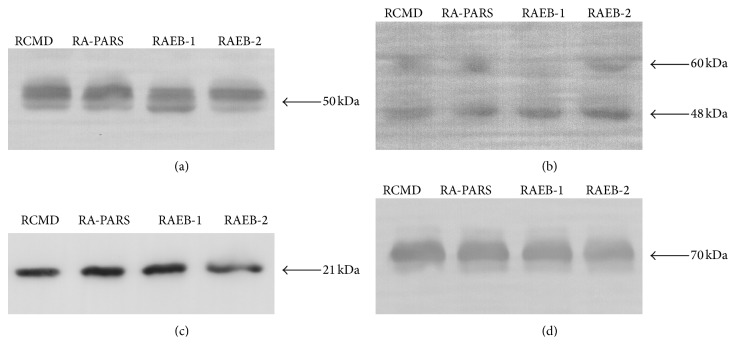
Western blot analysis. Western blot analysis was performed for alpha-2-HS-glycoprotein (a), leucine-rich alpha-2-glycoprotein (b), apolipoprotein A1 (c), and serum albumin (d) using pooled plasma samples of patients with four different MDS subgroups: refractory cytopenia with multilineage dysplasia (RCMD), refractory anemia or refractory anemia with ringed sideroblasts (RA-RARS), refractory anemia with excess blasts subtype 1 (RAEB-1), and refractory anemia with excess blasts subtype 2 (RAEB-2). For better clarity the western blot analysis results are shown as highlighted by brightness and contrast image adjustment.

In order to estimate whether the changes determined by 2D SDS-PAGE reflect the plasma level changes or posttranslational modifications of proteins, we performed western blot analysis for the selected proteins. Alpha-2-HS-glycoprotein and leucine-rich alpha-2-glycoprotein were selected as previously proposed MDS biomarker candidates (differentiating MDS subgroups from healthy controls), as well as due to their possible role in MDS pathophysiology as previously described in detail in [16] and [15], respectively. Apolipoprotein A1 was selected as it was identified in all four of the MDS subgroups studied and because it has been observed to form posttranslationally modified isoforms in cardiovascular disease patients [19, 22]. Serum albumin was selected as a control protein of the acute phase reaction to reflect a possible inflammation influence. Our findings are illustrated in Figure 2.

Western blotting of alpha-2-HS-glycoprotein showed several bands of approximately 50 kDa with a trend of decreasing intensity in advanced MDS subgroups. This result is in agreement with our electrophoretic data; however, it is apparent from the western blot that the representation of individual bands differs more substantially relative to total protein levels. The most obvious change can be observed for the bottom band, whose intensity increases substantially in the RAEB-1 subgroup. This supports the need for precise characterization of A2HSG posttranslational modifications and their quantification. Western blot analysis of leucine-rich alpha-2-glycoprotein revealed two bands of approximately 48 and 60 kDa. The lower 48 kDa band corresponds to that identified in 2D electrophoresis and shows the trend of increasing its intensity in advanced MDS subgroups. This is in agreement with the data obtained by 2D electrophoresis. As in the case of alpha-2-HS-glycoprotein, the modifications need to be characterized. Apolipoprotein A1 was shown to increase the spot volume in RA-RARS compared to RCMD and RAEB-2. It is clear from the western blot that the highest intensities were observed for both the RA-RARS and RAEB-1 subgroups. The fact that the changes were found for RA-RARS by 2D electrophoresis and not for RAEB-1 was most probably caused by the low number of RAEB-1 samples. No obvious changes were observed for albumin; therefore, we assume a minimal influence of the acute phase reaction on the results. Nevertheless, it has been recently shown that there are many other factors (genetic, clinical, or lifestyle factors) that can strongly affect protein plasma levels [23]. Moreover, the alterations in plasma protein levels may be affected by defects in cells' functions. For example, Blalock et al. [24] reported that phosphorylated form (on Thr451) of the dsRNA-dependent kinase accumulates in the cell nucleus of high-risk MDS patients and thus probably alters nuclear signaling. The implications of this finding on the disease or plasma protein changes are not known. A study by Aivado et al. [25] showed that CXC chemokine ligands 4 and 7 decreased their serum levels in advanced MDS patients compared to non-MDS cytopenia patients. The authors also showed that this serum decrease was related to platelets and, therefore, both the chemokines should be considered as platelet-derived markers. That platelet function impaired in MDS patients was recently confirmed in the study by Fröbel et al. [26]. In our study, the CXCL4 and CXCL7 were not identified; this is, however, not surprising when considering their low plasma (serum) levels. Aivado et al. used mass spectrometry-based detection which is capable of detecting proteins of lower concentrations compared to electrophoresis-based studies; the advantages and limitations of this approach have been reviewed in literature [27]. Unfortunately, there is no all-purpose proteomic method and a subset of proteins can be observed at once using a specific proteomic method. In the study by Chen et al. [28] ProteinChip array technology and mass spectrometry were used to investigate disease-associated and therapy-associated differences in sera of del(5q) MDS patients. Platelet factor 4 (also known as CXCL4) was found to be a potential therapy-associated marker; therefore, the results supported the observation by Aivado et al. [25]. Several proteins were proposed to be potential disease-associated markers (e.g., ITIH4, transferrin, transthyretin); these proteins were not identified in our reanalysis; however, it is not surprising as there were no non-MDS control samples used in our reanalysis contrary to the work by Chen et al. [28]. Nevertheless, when the results obtained by Chen et al. are compared to our previous protemic studies (which were used for this reanalysis) investigating different MDS subgroup patients with the healthy control samples, there were the same proteins identified. Since the proteins are altered between MDS and control samples but seem to be unaltered among MDS subgroups as shown in our reanalysis, these results indicate that the protein changes may be related to other (patho)physiological processes and not to be specific to MDS.

Identifying new biomarkers could have a significant impact on the clinical practice. While for diagnostics it is important to investigate the differences between the healthy (or nondisease) and patient cohorts, it is essential to identify alterations in the disease progression for prognosis and therapy monitoring. Considering MDS, it is important to describe the changes among different subgroups (as was the aim of our study), alterations related to the therapy (e.g., the study by Chen et al.), or specific changes during the disease progression. The changes related to MDS progression toward acute myeloid leukemia were investigated in work by Braoudaki et al. [29] in plasma, bone marrow, and cell lysate samples of pediatric patients. The most promising protein candidates observed in our reanalysis were also identified by Braoudaki et al.: leucine-rich alpha-2-glycoprotein and alpha-2-HS-glycoprotein. This observation further highlights the potential and importance of those protein candidates. Moreover, another protein coidentified in two spots (antithrombin-III) in our reanalysis was also observed in the study by Braoudaki et al.; antithrombin-III was found to be differentially expressed in both bone marrow and peripheral blood plasma samples and it was shown to be altered after 3 months of treatment. Thus, antithrombin-III could be another promising target of future proteomic investigations.

## 4. Conclusions

In conclusion, plasma protein biomarker candidates have been presented in this work with respect to different MDS subgroups. Alpha-2-HS-glycoprotein and leucine-rich alpha-2-glycoprotein appear to be the most promising candidates with regard to western blot observations, as well as our previous results detailing the differences in plasma proteome patterns between MDS subgroups and healthy donors. The presented results should be a catalyst for further MDS biomarker validation requiring precise protein posttranslational modification characterization, profiling of the changes in MDS subgroups, and extended statistical validation with large patient cohorts.

## Figures and Tables

**Table 1:** Patients' characteristics.

Patient	Sex	Age	Diagnosis	Karyotype	WBC [10^9^/L]	PLT [10^9^/L]	Blasts in PB [%]	NS [%]	IPSS	IPSS-R
1	f	21	RCMD	46, XX	3.51	31	0	22.9	Good	Very good
2	f	24	RCMD	46, XX	4.79	238	0	52	Good	Very good
3	f	29	RCMD	46, XX	3.93	19	0	27	Good	Very good
4	m	29	RCMD	46, XY-polyploidy	3.81	50	0	73	Good	Very good
5	f	30	RCMD	46, XX	2.53	134	0	36	Good	Very good
6	m	30	RCMD	46, XY	7.96	107	0	69	Good	Very good
7	m	49	RAEB2	46, XY	4.34	15	3	23	Good	Very good
8	f	50	RA	46, XX	3.64	184	0	59	Good	Very good
9	f	50	RCMD	46, XX, inv(9)	1.81	108	0	48	Intermediate	Intermediate
10	f	51	RCMD	46, XX, 9qh+	3.80	20	0	58	Intermediate	Intermediate
11	m	55	RCMD	46, XY	2.19	236	0	25	Good	Very good
12	f	56	RCMD	46, XX	3.90	129	0	39	Good	Very good
13	f	56	RCMD	46, XX	4.15	211	0	71	Good	Very good
14	m	58	RAEB1	45, XY, −18, multiple aberrations	1.26	28	0	30	Poor	Very poor
15	f	58	RAEB2	42~47, XX, del(5)(q?), −7, +8, der(12)t(7; 12)(?; p?13)ins(12; 7)(q?12; ?)ins(12; 7)(q?13; ?), der(17)t(17; 20)(p11.2; ?)	1.57	310	5.2	43.2	Poor	Very poor
16	m	58	RCMD	46, XY −46, XY, del(20)(q12)	2.00	211	0	56	Good	Good
17	m	58	RCMD	46, XY	2.48	153	0	55	Good	Very good
18	m	59	RAEB2	46, XY −43~44, XY, multiple changes	2.30	110	14	18	Poor	Very poor
19	m	59	RCMD	46, XY	2.81	103	0	50	Good	Very good
20	f	60	RA	46, XX	7.41	149	0	60	Good	Very good
21	m	60	RAEB1	46, XY	0.65	88	0	34	Good	Very good
22	f	60	RAEB2	46, XX	5.36	39	11	46	Good	Very good
23	m	61	RARS	46, XY	5.84	218	0	67	Good	Very good
24	m	62	RCMD	46, XY −45, X, −Y	6.82	89	0	65	Good	Very good
25	f	62	RCMD	46, XX	2.58	28	0	51	Good	Very good
26	m	62	RCMD	46, XY	4.49	56	0	61	Good	Very good
27	f	63	RA	46, XX −46, XX, del(5)(q13q13)	3.54	146	0	52	Good	Good
28	f	64	RAEB1	46, XX, t(2; 12)(p22; q13)	5.91	121	1	44	Intermediate	Intermediate
29	f	65	RA	46, XX −46, XX, del(5)(q15q33)	3.20	192	1	29	Good	Good
30	m	65	RCMD	46, XY −43~46, XY, der(2)t(2; 12)(q37; ?), del(11)(q13)	2.93	297	0	43	Poor	Very poor
31	m	66	RCMD	46, XY, 21ps+	6.74	81	0	52	Intermediate	Intermediate
32	f	66	RCMD	46, XX	2.79	16	0	84.3	Good	Very good
33	f	67	RAEB2	—	3.93	22	14	28	—	—
34	f	68	RAEB1	46, XX −46, XX, del(5)(q22q33)	3.80	412	0	50	Good	Good
35	m	68	RCMD	46, XY −45, X, −Y	19.94	399	3	51	Good	Very good
36	f	70	RAEB1	46, XX	1.13	150	0	46	Good	Very good
37	m	70	RAEB2	5q31 deletion (8 of 11 tests)	3.70	156	8	73	Good	Good
38	m	71	RAEB1	46, XY	4.25	85	3	17	Good	Very good
39	m	71	RAEB1	46, XY	1.39	21	0	20	Good	Very good
40	f	72	RCMD	46, XX, del(5)(q13.3q33.3)	4.18	119	0	47	Good	Good
41	f	76	RAEB2	47–51, XX-multiple changes	1.52	8	2	42	Poor	Very poor
42	m	78	RA	46, XY	8.65	162	0	75	Good	Very good
43	f	78	RA	46, XX, del(5)(q13q33)	3.77	288	0	67	Good	Good
44	m	78	RA	46, XY	5.36	243	1	68	Good	Very good
45	f	79	RAEB2	46, XX -46, XX, del(5)(q13q33)	0.43	6	38	8	Good	Good
46	f	86	RARS	46, XX	8.86	375	0	55	Good	Very good
47	m	89	RARS	46, XY	6.44	155	0	40	Good	Very good

WBC: white blood cells; PLT: platelets; PB: peripheral blood; NS: neutrophil segments.

**Table 2:** The numbers of spots found to differ between the compared pairs of MDS subgroups.

	RCMD	RA-RARS	RAEB-1	RAEB-2
RCMD	—	—	—	—
RA-RARS	6	—	—	—
RAEB-1	11	0	—	—
RAEB-2	6	5	0	—

**Table 3:** Brief characterization of the identified spots.

RCMD versus RA-RARS					
Increase in RCMD			Increase in RA-RARS		
Spot	*P*	*r*	Spot	*P*	*r*
8	0.00117	1.6	3	0.00002	2.3
15	0.00061	1.7	14	0.00534	1.6
—	—	—	17	0.00107	1.6
—	—	—	18	0.00093	1.6

RCMD versus RAEB-1					
Increase in RCMD			Increase in RAEB-1		
Spot	*P*	*r*	Spot	*P*	*r*

2	0.0032	1.8	1	0.00074	6.4
5	0.00382	1.6	12	0.00177	4.7
8	0.00011	2.0	13	0.00232	5.2
19	0.00124	1.6	14	0.00093	1.7
20	0.0035	1.5	16	0.00274	1.7
—	—	—	18	0.00519	1.6

RCMD versus RAEB-2					
Increase in RCMD			Increase in RAEB-2		
Spot	*P*	*r*	Spot	*P*	*r*

8	0.00392	1.6	6	0.00787	1.6
9	0.00335	1.6	7	0.00525	1.7
—	—	—	10	0.00659	1.6
—	—	—	18	0.00701	1.6

RA-RARS versus RAEB-2					
Increase in RA-RARS			Increase in RAEB-2		
Spot	*P*	*r*	Spot	*P*	*r*

3	0.00538	2.1	10	0.00435	1.8
4	0.00348	1.5	11	0.00104	1.6
9	0.00008	1.8	—	—	—

*P*: *t*-test *P* value, *r*: fold change value.

**Table 4:** Protein identification.

Spot	Protein identification	Peptides	AN	SC (%)
1	Alpha-1-antitrypsin	12	P01009	38

2	Alpha-2-HS-glycoprotein	2	P02765	13

3	Apolipoprotein A-I	6	P02647	25

4	Apolipoprotein A-IV	10	P06727	44

5	Hemopexin	3	P02790	11

6	Leucine-rich alpha-2-glycoprotein	4	P02750	23

7	Leucine-rich alpha-2-glycoprotein	3	P02750	18

8	Retinol-binding protein 4	2	P02753	16

9	Actin, cytoplasmic 1; 2	4; 4	P60709; P63261	22; 22
	Apolipoprotein A-IV	5	P06727	21

10	Alpha-1-antichymotrypsin	6	P01011	23
	Plasma protease C1 inhibitor	4	P05155	13

11	Alpha-1-antichymotrypsin	4	P01011	14
	Plasma protease C1 inhibitor	4	P05155	18

12	Alpha-1-antitrypsin	10	P01009	29
	Antithrombin-III	2	P01008	8

13	Alpha-1-antitrypsin	9	P01009	28
	Antithrombin-III	2	P01008	8

14	Ig mu chain C region	2	P01871	17
	Prothrombin	3	P00734	20

15	Plasma protease C1 inhibitor	3	P05155	13
	Alpha-1-antichymotrypsin	2	P01011	10

16	Alpha-1-antitrypsin	3	P01009	24
	Prothrombin	4	P00734	24
	Complement C4-A; B	4	P0C0L4; P0C0L5	4

17	Prothrombin	3	P00734	25
	Serum albumin	3	P02768	11
	Ig mu chain C region	2	P01871	13

18	Serum albumin	3	P02768	8
	Ig mu chain C region	2	P01871	19
	Prothrombin	3	P00734	27

19	Alpha-1-antichymotrypsin	3	P01011	11
	Alpha-2-HS-glycoprotein	2	P02765	9
	Kininogen-1	3	P01042	8
	Corticosteroid-binding globulin	2	P08185	14

20	Pigment epithelium-derived factor	4	P36955	16
	Complement factor I	4	P05156	10
	Beta-2-glycoprotein 1	3	P02749	26
	Alpha-1-antichymotrypsin	2	P01011	14

AN: protein accession number (UniProt), SC: sequence coverage in %.
